# Optimization for Starve Fed/Flood Fed Single Screw Extrusion of Polymeric Materials

**DOI:** 10.3390/polym12010149

**Published:** 2020-01-07

**Authors:** Andrzej Nastaj, Krzysztof Wilczyński

**Affiliations:** Polymer Processing Department, Faculty of Production Engineering, Warsaw University of Technology, Narbutta 85, 02-524 Warsaw, Poland; a.nastaj@wip.pw.edu.pl

**Keywords:** polymers, extrusion, optimization

## Abstract

A novel computer optimization system for flood fed/starve fed single screw extrusion of polymeric materials has been developed. This coupled system allows us to optimize single screw extrusion both flood fed and starve fed. Optimization is based on process simulation which is performed using global extrusion model GSEM (Global Screw Extrusion Model). The process is optimized with the use of GASEO (Genetic Algorithms Screw Extrusion Optimization) procedures which were developed using Genetic Algorithms. An example of optimization of extrusion process parameters has been presented to maximize extrusion output and minimize specific energy consumption. Optimization has been performed in a unique and original way in a coupled manner when both modes of feeding were allowed. The studies have shown that the optimal process is extrusion with starving. In this case, the global objective function reached the highest value, and extrusion throughput was relatively high and specific energy consumption was minimal.

## 1. Introduction

The design of polymer processing is currently supported by computer simulations based on the mathematical models of manufacturing processes. Computer modeling allows us to predict the process course on the basis of process parameters (materials, geometry, operating). The process, e.g., extrusion, can be characterized by throughput, pressure/temperature distribution, power consumption, etc. Thus, it is possible to predict the output process parameters based on the input data.

Process computer modeling, however, does not solve the inverse problem of determining the process input parameters to obtain the optimal output parameters. Typically, the models do not allow us to select the process parameters that meet the production goals, e.g., maximizing process output, or minimizing energy consumption. Thus, these do not allow us to optimize the process according to the specific optimization criteria.

Extrusion is the most massive and important technology in the polymer processing industry. It is widely used for the production of film, sheet, pipe, and profiles and for specialty processing operations, such as compounding, mixing, granulating, etc. Optimization of an extrusion is a conflicting, multi-objective problem. It is complicated by the number of variables (materials, geometry, operating) and non-linear relations, and by opposing criteria, e.g., throughput and power consumption. It is difficult to find a global optimum avoiding local optima.

There are two approaches to solve the optimization problem, by experiment or simulations. Optimization started with experiments using various screws and operating data. The optimum was simply the best one tried. This was time-consuming, expensive, and nothing warranted that global optimum was found.

Statistics, regression, and response surface analyses may be used for optimization both by experiment and simulations. Underwood [1] first used factorial design of experiments to optimize extrusion. The optimum was found by locating the extremum on the response surfaces relating the input and output variables. Later, other researches applied this approach, e.g., Verbraak and Meijer [2]. The main drawback of this was the number of experiments required.

Thus, using process modeling seemed to be more efficient. First trials of optimization by simulation were performed by Tadmor and Klein [3], Maddock and Smith [4], as well as Helmy and Parnaby [5]. These were limited to conventional screws.

Later, more sophisticated models were developed. Potente and Krell [6] developed a strategy for screw optimization by means of DOE (Design of Experiments) and multiple regression using a global extrusion model REX [7,8,9]. Thibodeau and Lafleur [10,11] used STATISTICA software to locate the optimum on a response surface given by the model of Ecole Polytechnique de Montreal [12,13]. Statistical methods were also used by the authors [14] who applied SSEM (Single Screw Extrusion Model) program [15,16]. The main drawback of statistical approach was the number of simulations required in the response surface of high data density and a danger of finding local optima, rather than a global one.

Artificial intelligence, including neural networks, Genetic Algorithms and fuzzy systems, may be also considered for modeling and optimization of manufacturing processes. These provide continuous or discrete solutions, and the learning process using available data is included. Fundamental research using Genetic Algorithms was performed by Covas and Gaspar-Cunha who first developed optimization procedures for single screw extrusion [17,18,19], and co-rotating twin screw extrusion [20,21,22]. The authors made optimization attempts using neural networks [23] and Genetic Algorithms [24,25]. Recently, Genetic Algorithms were used for extrusion scale-up [26,27] and for optimization of injection molding [28,29].

Genetic Algorithms (or evolution techniques) comparing to other optimization methods have the following features:

- Parameters of optimization task are processed not directly, but in the coded form;
- Searching of solution is performed from a certain population, and not from one point, which means the probability of getting stuck in the local extreme is less;
- Selection rules are rather probabilistic than deterministic;
- A new search area of the expected higher quality is defined using previous experiences, thanks to which, despite some randomness, they do not amount to accidental wandering;
- The objective function is only used, and not its derivatives.

Up to date, there is a lack of optimization studies on single screw extrusion with metered feeding (starve fed extrusion) and counter-rotating twin screw extrusion, although the mathematical models of these global models for starve fed single screw extrusion [30,31,32] and global models for counter-rotating extrusion [33,34,35,36,37] have been recently developed.

In this paper, a novel computer optimization system for flood fed/starve fed single screw extrusion of polymeric materials has been presented. This coupled system allows to optimize single screw extrusion both flood fed and starve fed. Optimization is based on process simulation, which is performed using global extrusion model GSEM (Global Screw Extrusion Model) [30,31,32]. The process is optimized using GASEO (Genetic Algorithms Screw Extrusion Optinization) procedures which are based on Genetic Algorithms. An example of optimization of process parameters has been presented to maximize extrusion output and minimize specific energy consumption.

## 2. Flood Fed vs. Starve Fed Extrusion

**Figure 1:**
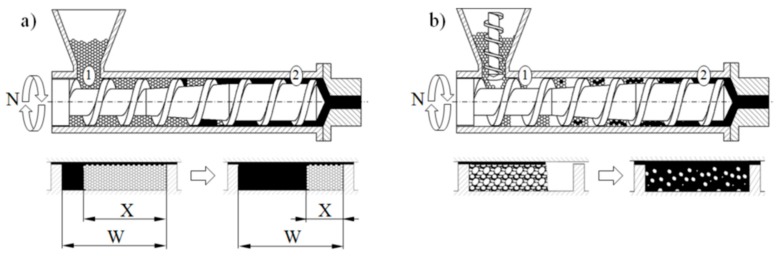
Melting models for single screw extrusion: (**a**) flood fed extrusion, (**b**) starve fed extrusion, (1)—solid conveying section, (2)—melt conveying section, X—solid bed width, W—screw channel width [32].

Single screw extruders can be flood fed or starve fed. When flood feeding, the screw takes in as much material as it can handle, and the bottom section of the hopper is fully filled with material. When starve feeding, the material is metered into the machine with a feeder, and the bottom section of the hopper is not fully filled with material. It is depicted in Figure 1.

Starve fed extrusion has several advantages over the flood fed extrusion, e.g., [38,39,40,41,42,43]. The pressure built up along the screw is lower, and there is less chance of agglomeration, and mixing action may be improved. Melting action is faster since the pellets are not compacted into a dense solid bed, and the polymer granules maintain their individuality as melting progresses. In starve fed extrusion, the screw speed can be varied at constant output, as well as the output can be varied at constant screw speed, and there is a greater degree of process control. Generally, starve fed extruders may be used for more demanding operations, although there are some disadvantages. The extruder throughput is reduced below its capacity, and the operation is more complicated in that an external device is necessary to feed the material into the machine.

Although the flood fed single screw extrusion was broadly investigated and modeled, little information was presented on the starve fed extrusion. The state-of-the-art for modeling of extrusion was presented in some fundamental books, e.g., by Rauwendaal [43], White and Potente [44], Tadmor and Gogos [45], Osswald and Hernandez-Ortiz [46], and Agassant et al. [47], as well as in some papers, e.g., [48,49,50,51,52]. Wilczyński et al. summarized and discussed this in a review paper [53].

Recently, Wilczyński et al. [54,55] based on experimental studies proposed the melting mechanism and melting model for the starve fed single screw extrusion, and then developed the first computer model of this process, SSEM-Starve [30]. According to this model, two stages of melting are distinguished: melting by conduction in the partially filled region of the screw where the polymer granules are collected at the active screw flight and melting by energy dissipation in the fully filled region where the unmolten solid particles are suspended in the previously molten material (Figure 1b). This basic model was later extended to non-conventional screw configurations [31,32] and extrusion of polymer blends and composites [56,57,58,59].

## 3. Modeling of Extrusion

Extrusion is a continuous process of co-operation of the extruder and the die. The flow phenomena occurring in the extruder determine the flow in the extrusion die, and vice versa, the flow in the extrusion die determines the phenomena occurring in the extruder. Thus, any change of processing conditions in the extruder causes a change of processing conditions in the die, and vice versa. Extrusion modeling cannot be limited to the modeling of flow in the extruder and has to include the modeling of flow in the extrusion die.

**Figure 2:**
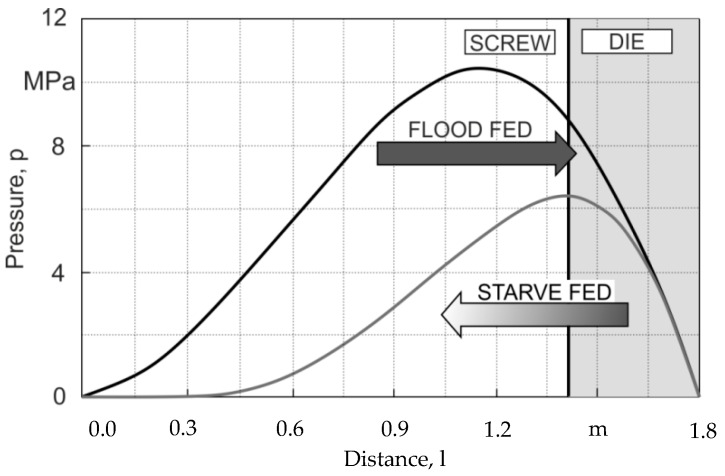
A forward scheme of computations for flood fed extrusion and a backward scheme of computations for starve fed extrusion [53].

The term global modeling means modeling of the interacting phenomena occurring in the extruder and the die, that is, modeling of the extruder/die system (screw/die system). This requires the use of computation algorithm appropriate for a given type of extrusion. For flood fed extrusion, the forward scheme of computation is applied. In this case, the material flow rate is not known, and is the result of the screw/die co-operation and must be determined in multiple iterative computations. For starve fed extrusion, the backward scheme of computation is used, i.e., the inverse computation algorithm since the pressure profile is not continuous here, and there is no continuous the flow rate/pressure relation. In this case, the flow rate is known and equal to the flow rate of the material metered by the dosing device (Figure 2).

Simulation schemes for flood fed single screw extrusion are relatively well known, e.g., [8,19,60,61]. In this case, the modeling proceeds from the hopper to the die according to the forward scheme of computations (Figure 2), and the extrusion operating point is searched which defines the extrusion flow rate and die pressure. The flow rate is not known and results from the screw/die co-operation. Computations start for some presumed flow rate, e.g., equal to the drag flow rate and solid conveying, melting, melt conveying, and die flow are simulated. The calculated pressure at the die exit is compared to the atmospheric pressure, and the computation is achieved when both pressures are equal. Otherwise, the presumed flow rate is modified, and computations are iteratively repeated until the convergence is reached.

Simulation schemes for starve fed single screw extrusion are much less known, e.g., [30,31,32]. In this case, the modeling requires an inverse approach. The flow rate is known and is equal to the feeding rate. In this case, the die pressure is computed first for some presumed polymer melt temperature. Then, the pressure gradient back along the screw is calculated using the screw pumping characteristics. When the pressure falls to zero, the starvation begins, and the screw filling is computed. The calculated temperature at the end of melting is compared to the melting point, and the computation is achieved when both temperatures are equal. Otherwise, the presumed melt temperature is modified, and computations are iteratively repeated until the convergence is reached. When second stage of melting appears, the computation scheme gets much more complicated since the location of the transition partially/fully filled screw has to be determined.

Using the inverse computation approach, the authors developed the global models for closely intermeshing counter-rotating twin screw extruders [33,34,37], as have done other researchers for co-rotating twin screw extruders [62,63,64,65]. However, those composite models using one-stage melting models were much simpler in execution. Moreover, the location of the melting regions was not computed but specified a-priori in those cases.

**Figure 3:**
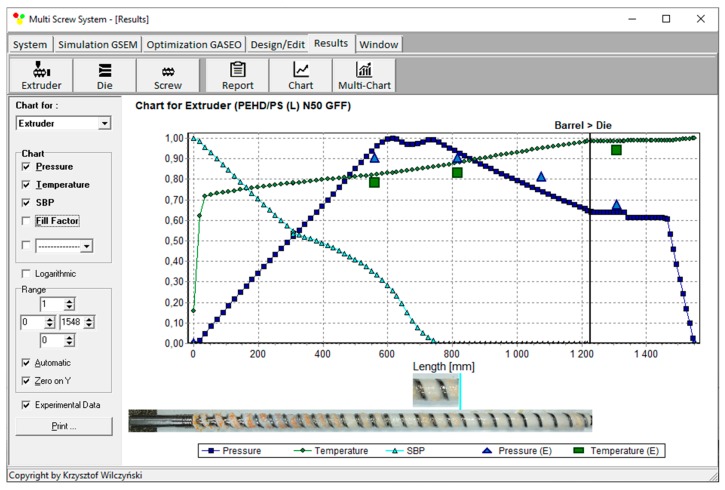
Overall extrusion characteristics, simulation, and experimentation data using Global Screw Extrusion Model (GSEM) model; flood fed single screw extrusion (material and process data (see Section 5.1), screw speed *N* = 50 rpm, length of metering section *L*_M_ = 590 mm): SBP—solid bed profile, *E*—experiment.

**Figure 4:**
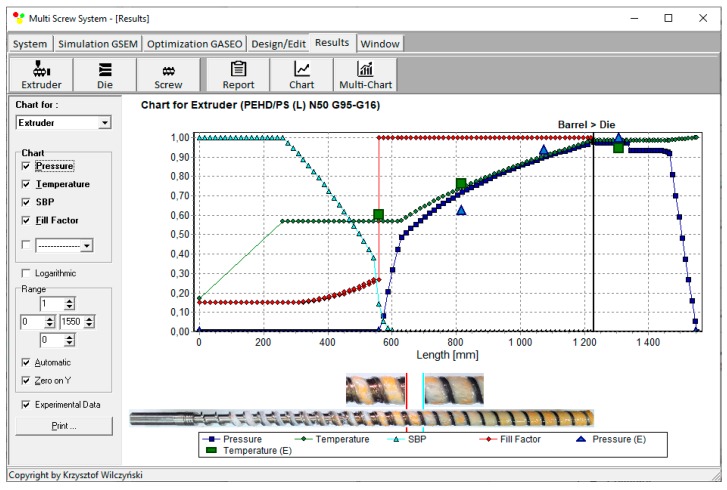
Overall extrusion characteristics, simulation, and experimentation data using GSEM model; starve fed single screw extrusion (material and process data (see Section 5.1), Q = 16 kg/h, screw speed *N* = 50 rpm, length of metering section *L*_M_ = 590 mm): SBP—solid bed profile, *E*—experiment.

Recently, the authors developed GSEM (Global Screw Extrusion Model) program which allows us to simulate the starve fed/flood single screw extrusion [31,32]. Examples of simulations are depicted in Figure 3 and Figure 4. These simulations were validated by experiment, and predictions were good. Overall process characteristics (dimensionless) are presented, which include pressure and temperature profiles, as well as a profile of melting progress. In addition, in the case of starve fed extrusion, a profile of screw filling is shown. It is clearly seen (Figure 4) that the pressure falls to zero when starvation begins. Two stages of melting can be also observed. Starve fed region and fully filled region are also clearly seen on the validation pictures.

## 4. Optimization Procedure

An optimization program GASEO (Genetic Algorithms Screw Extrusion Optimization) has been developed which is based on Genetic Algorithms. The source of data for optimization is process simulations made by using GSEM (Global Screw Extrusion Model) program.

**Figure 5:**
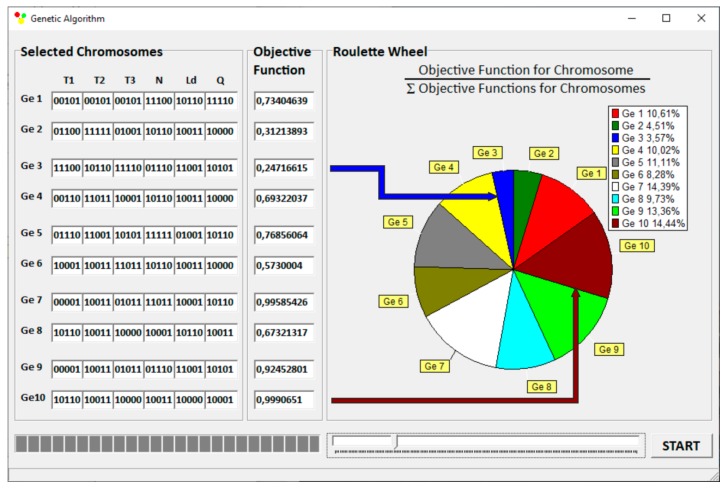
Execution of Genetic Algorithms: selection of initial population and evaluation of chromosome adaptation.

GASEO optimization program, in cooperation with GSEM simulation program, makes it possible to carry out extrusion optimization with any number of optimized variables, with various process optimization criteria, e.g., extrusion output or power consumption. Accuracy of searching the response surface is determined by the number of divisions of data range which results from the length of writing these numbers in a binary form. In GASEO program, the length of binary series is adjustable, and its maximum length is 255 characters. This allows the range of each variable to be divided into 2^255^ values. A “roulette wheel” method is used for selection, which is relatively simple in software implementation. The condition for optimization stopping is 100 times the best data set to occur. A scheme of operation of “roulette wheel” is depicted in Figure 5. The surfaces of “roulette wheel” assigned to individual genotypes are proportional here to the values of objective functions generated by these genotypes. For example, the Ge10 genotype generates the highest value of the objective function *F*_i_ = 0.9991 and covers an area on the “roulette wheel” equal to 14.44% of the total area of this. However, the Ge3 genotype generates the smallest value of the objective function *F*_i_ = 0.2472, covering an area equal to 3.57% of the total area of the “roulette wheel”.

**Figure 6:**
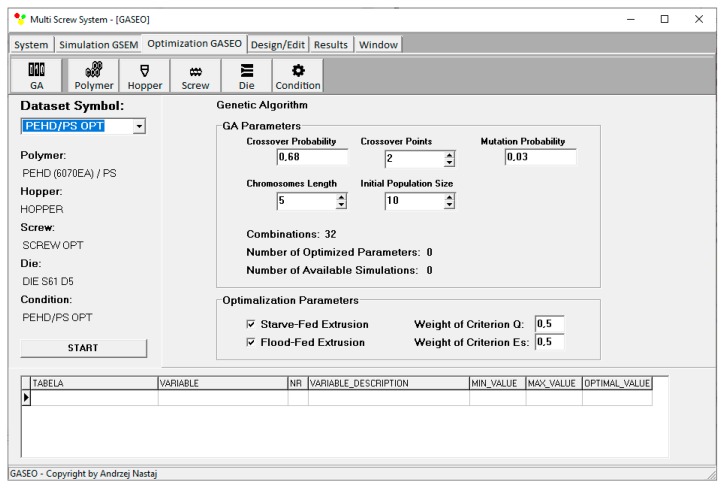
Genetic Algorithms’ parameters defining optimization procedure.

Optimization procedure is defined by Genetic Algorithms’ parameters, number of optimized parameters, initial population size, chromosomes length, crossover probability, crossover points, and mutation probability. Optimization can be carried out selectively for flood fed extrusion or starve fed extrusion but can also be carried out in a unique way in a coupled manner when both modes of feeding are allowed. This is depicted in Figure 6.

## 5. Optimization

### 5.1. Research Program

Optimization was carried out for single screw extrusion of polyblend composed of high-density polyethylene (HDPE, 85%) and polystyrene (PS, 15%). This immiscible blend [66] was the subject of our recent extrusion studies both experimental and modeling [57,59].

In the present optimization studies, conventional three-sectional screw of diameter *D* = 45 mm, and length/diameter ratio *L*/*D* = 27 was used as an initial screw geometry configuration. It has feeding, compression, and metering sections with length/diameter ratios equal to (*L*/*D*)_F_ = 10.78, (*L*/*D*)_C_ = 7.11, and (*L*/*D*)_M_ = 9.11. The compression ratio, which is defined as a ratio of the channel depth (*H*_F_) in the feeding section to the channel depth in the metering section (*H*_M_), i.e., CR = *H*_F_/*H*_M_, was equal to CR = 2.66 (*H*_F_ = 8 mm, *H*_M_ = 3 mm). The die for extruding rods of diameter *D* = 5 mm was applied.

Flood fed extrusion and starve fed extrusion were considered. HDPE Rigidex 6070EA (manufactured by BP Chemicals) and PS Styrolution 158N (manufactured by INEOS Styrolution Group) were used. HDPE has density of 0.952 g/cm^3^ (solid) and 0.722 g/cm^3^ (melt) and Melt Flow Rate MFR = 7.6 g/10 min (190 °C, 2.16 kg). PS has density of 1.050 g/cm^3^ (solid) and 0.936 g/cm^3^ (melt) and Melt Volume Rate MVR = 3.0 cm^3^/10 min (200 °C, 5 kg).

Rheological flow properties of HDPE/PS polyblend were determined on the basis of capillary rheometry using high-pressure rheometer RG-25 (from Goettfert, Buchen, Germany), and were modeled using Klein equation

(1) $$ln\eta =A_{0}+A_{1}ln\dot{\gamma }+A_{11}ln^{2}\dot{\gamma }+A_{12}Tln^{2}\dot{\gamma }+A_{2}T+A_{22}T^{2}$$

where η is the viscosity; $\dot{\gamma }$ is the shear rate, T is the temperature; *A*_0_, *A*_1_, *A*_11_, *A*_12_, *A*_2_, *A*_22_ are the model parameters, *A*_0_ = 12.1778, *A*_1_ = −0.3109, *A*_11_ = −0.0348, *A*_12_ = 0.0012, *A*_2_ = −0.0289, *A*_22_ = 0.00002798.

Optimization was carried out both for flood fed extrusion and starve fed extrusion to maximize extrusion output *Q*_max_ (kg/h) and to minimize specific energy consumption *E*_s min_ (kJ/kg). The optimized parameters were screw speed, barrel temperatures and length of screw metering section, which are collected in Table 1, where the range of these is also shown. The range of optimized parameters was established on the basis of experimental and simulation studies. The upper value of flow rate was equal to the value of flow rate obtained during flood fed extrusion at the screw speed *N*_max_ = 120 rpm. The values of parameters for computation the global objective function were selected randomly. The starve fed or flood fed mode of feeding was also selected randomly.

A global objective function was defined as

(2) $$F_{i}=w_{Q}\cdot Q_{i{\_}_{norm}}+w_{Es}\cdot E_{s\;i\_norm}$$

where *F*_i_ is the global objective function, *Q*_i_norm_ is the normalized flow rate, *w*_Q_ is the weight of flow rate, *E*_s i_norm_ is the normalized specific energy consumption, *w*_Es_ is the weight of specific energy consumption, *i* is the number of next value from the data set.

The output parameters (optimization criteria) were normalized as

(3) $$Q_{i\_norm}=\frac{Q_{i}-Q_{min}}{Q_{max}-Q_{min}}$$

(4) $$E_{s\;i\_norm}=\frac{E_{s\;max}-E_{s\;i}}{E_{s\;max}-E_{s\;min}}$$

### 5.2. Results

Optimization has been carried out in a unique way in a coupled manner when both modes of feeding are allowed.

Results of optimization are presented for various weights of optimization criteria in Table 2. Three sets of optimal parameters have been obtained for various criteria weights (Table 2, results 1–3, 4–6, and 7–9). It is worth noting that the optimization indicated extrusion with starving to be optimal. In this case, the global objective function reached the highest value. Extrusion throughput was relatively high and specific energy consumption was minimal.

**Figure 7:**
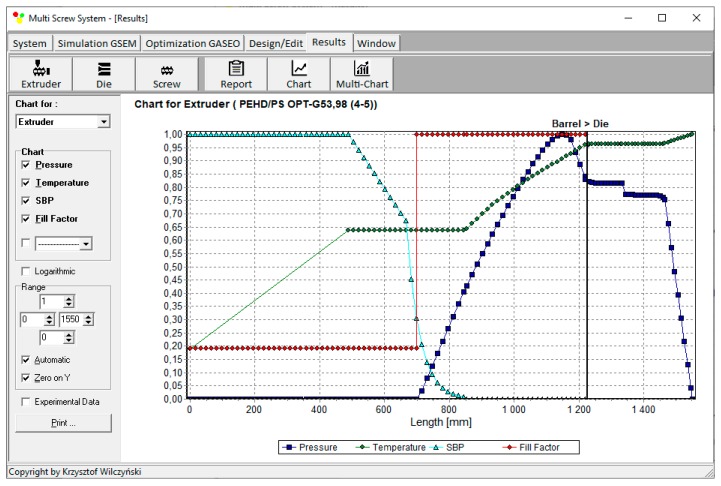
Overall process characteristics for starve fed single screw extrusion for weights of optimization criteria equal to *w*_Q_ = 0.5, *w*_Es_ = 0.5.

**Figure 8:**
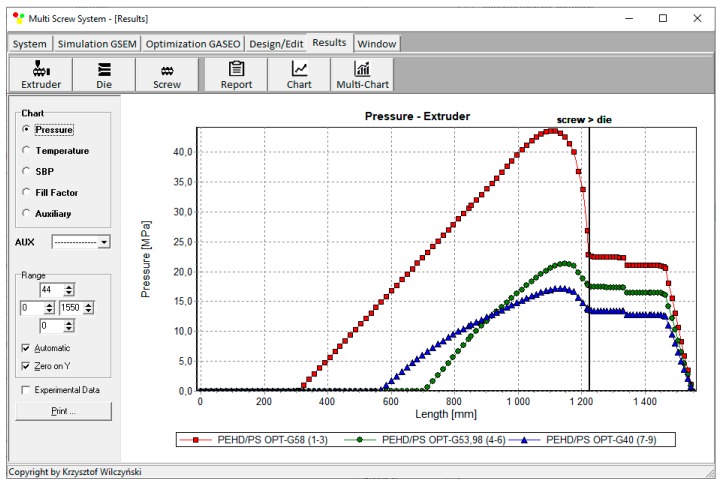
Pressure profiles at optimal process parameters for various weights of optimization criteria, *w*_Q_ and *w*_Es_ (Table 2, results 1–3, 4–6, 7–9).

**Figure 9:**
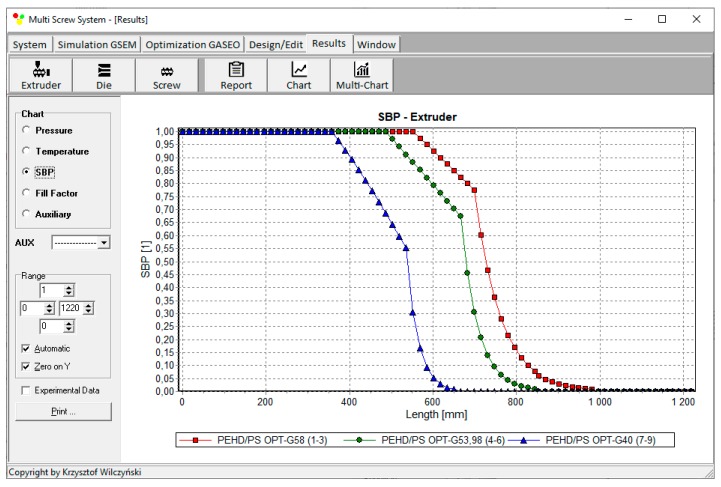
Solid bed profiles (SBP) at optimal process parameters for various weights of optimization criteria, *w*_Q_ and *w*_Es_ (Table 2, results 1–3, 4–6, 7–9).

**Figure 10:**
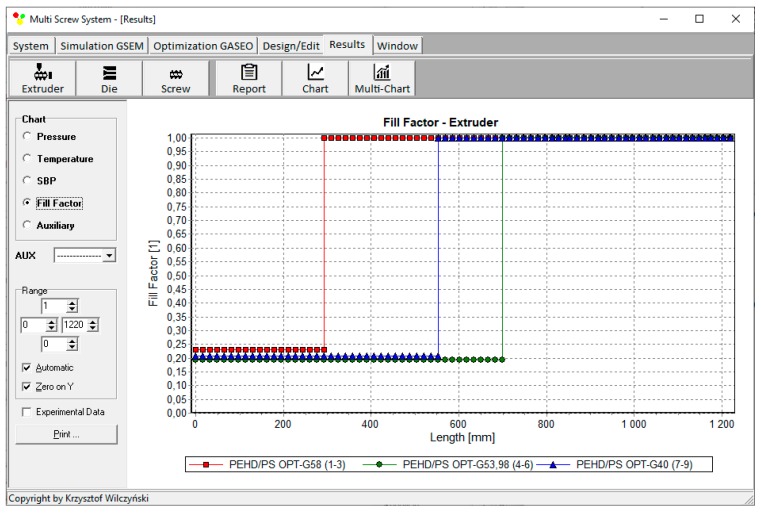
Filling factor profiles at optimal process parameters for various weights of optimization criteria, *w*_Q_ and *w*_Es_ (Table 2, results 1–3, 4–6, 7–9).

Process simulations have been performed for each set of optimal parameters. Overall process characteristics obtained at the optimal parameters for medium values of weights of optimization criteria, *w*_Q_ = 0.5, *w*_Es_ = 0.5 (Table 2, results 5), are depicted in Figure 7. Simulations at optimal process parameters for various weights of optimization criteria are presented in Figure 8, Figure 9 and Figure 10. Pressure profiles, depicted in Figure 8, clearly show that when flow rate is preferred, i.e., *w*_Q_ > *w*_Es_, pressure substantially increases, and when specific energy consumption is preferred, i.e., *w*_Q_ < *w*_Es_, pressure decreases. It results from Figure 9 that when flow rate is preferred, i.e., *w*_Q_ > *w*_Es_, melting is slower, and opposite, when specific energy consumption is preferred, i.e., *w*_Q_ < *w*_Es_, melting is faster. Two stages of melting can be also observed in all simulation cases. Screw filling profiles are depicted in Figure 10. It is clearly seen that when starvation begins, the pressure falls to zero (compare to Figure 8).

In Table 3, the highest values of global objective function obtained for various weights of optimization criteria, in the case of flood fed mode of feeding, are presented. Three sets of parameters have been obtained for various criteria weights (Table 3, results 1–4, 5, and 6–9). The values of global objective function are in each case smaller than in the case of starve fed mode of feeding (see Table 2).

**Figure 11:**
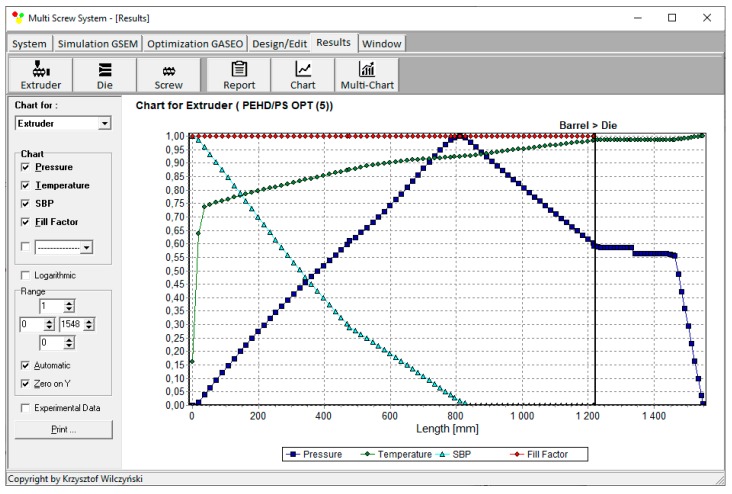
Overall process characteristics for flood fed single screw extrusion for weights of optimization criteria equal to *w*_Q_ = 0.5, *w*_Es_ = 0.5.

**Figure 12:**
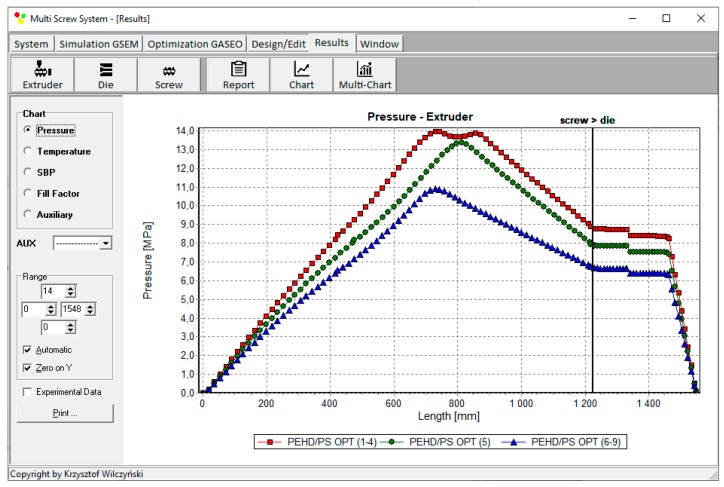
Pressure profiles at optimal process parameters for various weights of optimization criteria, *w*_Q_ and *w*_Es_ (Table 3, results 1–4, 5, 6–9).

**Figure 13:**
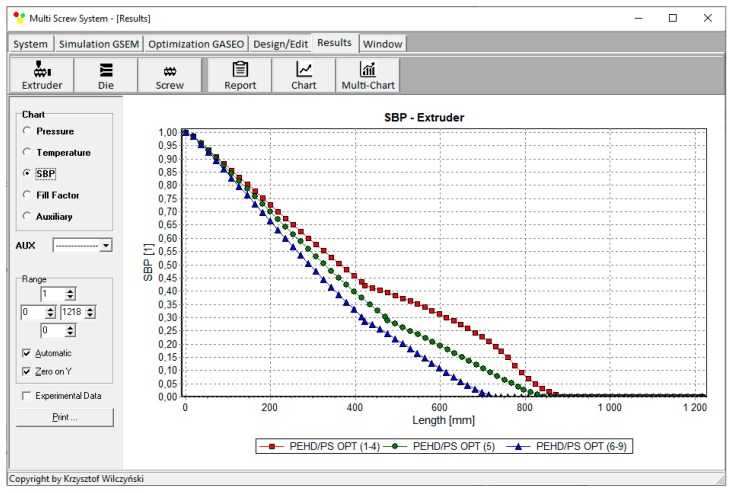
Solid bed profiles (SBP) at optimal process parameters for various weights of optimization criteria, *w*_Q_ and *w*_Es_ (Table 3, results 1–4, 5, 6–9).

Process simulations have been performed for each set of these parameters. Overall process characteristics obtained for medium values of weights of optimization criteria, *w*_Q_ = 0.5, *w*_Es_ = 0.5 (Table 3, results 5), are depicted in Figure 11. Screw filling profile clearly shows that when starvation is not allowed, the pressure does not fall to zero (compare to Figure 7). Simulations for various weights of optimization criteria are presented in Figure 12 and Figure 13. Pressure profiles, depicted in Figure 12, clearly show that when flow rate is preferred, i.e., *w*_Q_ > *w*_Es_, pressure increases, and when specific energy consumption is preferred, i.e., *w*_Q_ < *w*_Es_, pressure decreases. It results from Figure 13 that when flow rate is preferred, i.e., *w*_Q_ > *w*_Es_, melting is slower, and opposite, when specific energy consumption is preferred, i.e., *w*_Q_ < *w*_Es_, melting is faster.

**Figure 14:**
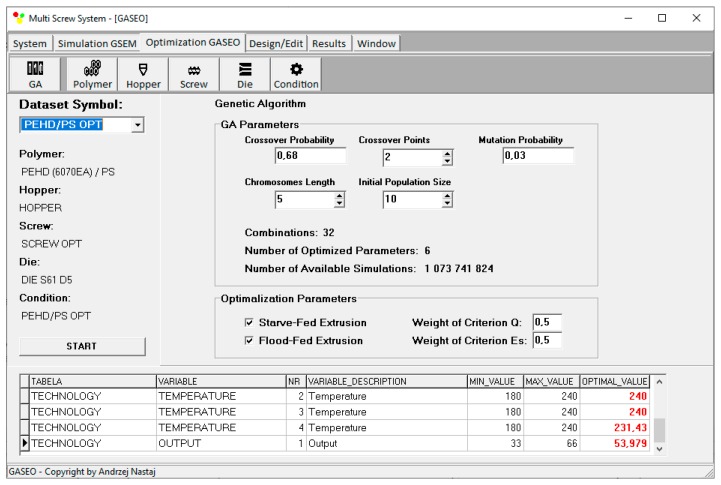
Optimization results for starve fed extrusion for weights of optimization criteria equal to *w*_Q_ = 0.5, *w*_Es_ = 0.5.

An example of results of optimization (for criteria weights *w*_Q_ = 0.5, *w*_Es_ = 0.5) is presented on the screen of GASEO program, which is depicted in Figure 14. The parameters of optimization are also seen, as well as the values of optimal parameters.

## 6. Conclusions

A novel computer optimization system for flood fed/starve fed single screw extrusion of polymeric materials has been developed. This coupled system allows to optimize single screw extrusion both flood fed and starve fed. Optimization is based on process simulation which is performed using global extrusion model GSEM (Global Screw Extrusion Model). The process is optimized using GASEO (Genetic Algorithms Screw Extrusion Model) procedures which are based on Genetic Algorithms. An example of optimization of process parameters has been presented to maximize extrusion output and minimize specific energy consumption.

It is worth noting that the optimization indicated extrusion with starving to be optimal. In this case, the global objective function reached the highest value. Extrusion throughput was relatively high and specific energy consumption was minimal.

Process simulations have shown that when flow rate is preferred, i.e., *w*_Q_ > *w*_Es_, pressure increases, and melting is slower. However, when specific energy consumption is preferred, i.e., *w*_Q_ < *w*_Es_, pressure decreases, and melting is faster. Simulations have also shown that when starvation begins, the pressure falls to zero.

## Figures and Tables

**Table 1:** Research program.

Screw Speed *N*, rpm	Length of Screw Metering Section *L*_d_, mm	Barrel Temperature *T*_I_, °C	Barrel Temperature *T*_II_, °C	Barrel Temperature *T*_III_, °C	Barrel Temperature *T*_IV_, °C	Flow Rate Q, kg/h
40 ÷ 120	45 ÷ 853	150	180 ÷ 240	180 ÷ 240	180 ÷ 240	0 ÷ 66

**Table 2:** Maximum values of global objective functions at selected weights of optimization criteria, flow rate and specific energy consumption.

	Weight of Criterion								
	*w*_Q_ = 0.9 *w*_Es_ = 0.1	*w*_Q_ = 0.8 *w*_Es_ = 0.2	*w*_Q_ = 0.7 *w*_Es_ = 0.3	*w*_Q_ = 0.6 *w*_Es_ = 0.4	*w*_Q_ = 0.5 *w*_Es_ = 0.5	*w*_Q_ = 0.4 *w*_Es_ = 0.6	*w*_Q_ = 0.3 *w*_Es_ = 0.7	*w*_Q_ = 0.2 *w*_Es_ = 0.8	*w*_Q_ = 0.1 *w*_Es_ = 0.9
No	1	2	3	4	5	6	7	8	9
Screw speed *N*, rpm	120	120	120	120	120	120	74	74	74
Lenght of screw metering section *L*_d_, mm	45	45	45	45	45	45	45	45	45
Barrel temperature *T*_II_, °C	240	240	240	240	240	240	240	240	240
Barrel temperature *T*_III_, °C	240	240	240	240	240	240	240	240	240
Barrel temperature *T*_IV_, °C	231	231	231	231	231	231	231	231	231
Flow rate Q, kg/h	58	58	58	53.98	53.98	53.98	40	40	40
Specific Energy consumption *E*_s_, kJ/kg	525.22	525.22	525.22	403.5	403.5	403.5	262.67	262.67	262.67
Starve/Flood	Starve	Starve	Starve	Starve	Starve	Starve	Starve	Starve	Starve
Objective function *F*_i_	0.9643	0.9286	0.8929	0.8700	0.8593	0.8576	0.8789	0.9174	0.9560

**Table 3:** Maximum values of global objective functions at selected weights of optimization criteria, flow rate and specific energy consumption (flood fed mode of feeding).

	Weight of Criterion								
	*w*_Q_ = 0.9 *w*_Es_ = 0.1	*w*_Q_ = 0.8 *w*_Es_ = 0.2	*w*_Q_ = 0.7 *w*_Es_ = 0.3	*w*_Q_ = 0.6 *w*_Es_ = 0.4	*w*_Q_ = 0.5 *w*_Es_ = 0.5	*w*_Q_ = 0.4 *w*_Es_ = 0.6	*w*_Q_ = 0.3 *w*_Es_ = 0.7	*w*_Q_ = 0.2 *w*_Es_ = 0.8	*w*_Q_ = 0.1 *w*_Es_ = 0.9
No	1	2	3	4	5	6	7	8	9
Screw speed *N*, rpm	63	63	63	63	51	40	40	40	40
Lenght of screw metering section *L*_d_, mm	475.65	475.65	475.65	475.65	422.1	475.65	475.65	475.65	475.65
Barrel temperature *T*_II_, °C	189	189	189	189	214	223	223	223	223
Barrel temperature *T*_III_, °C	180	180	180	180	180	197	197	197	197
Barrel temperature *T*_IV_, °C	180	180	180	180	189	180	180	180	180
Flow rate Q, kg/h	23.24	23.24	23.24	23.24	18.85	14.66	14.66	14.66	14.66
Specific Energy consumption *E*_s_, kJ/kg	412.75	412.75	412.75	412.75	322.02	258.64	258.64	258.64	258.64
Starve/Flood	Flood	Flood	Flood	Flood	Flood	Flood	Flood	Flood	Flood
Objective function *F*_i_	0.2996	0.3545	0.4094	0.4643	0.5322	0.6233	0.7175	0.8116	0.9058
